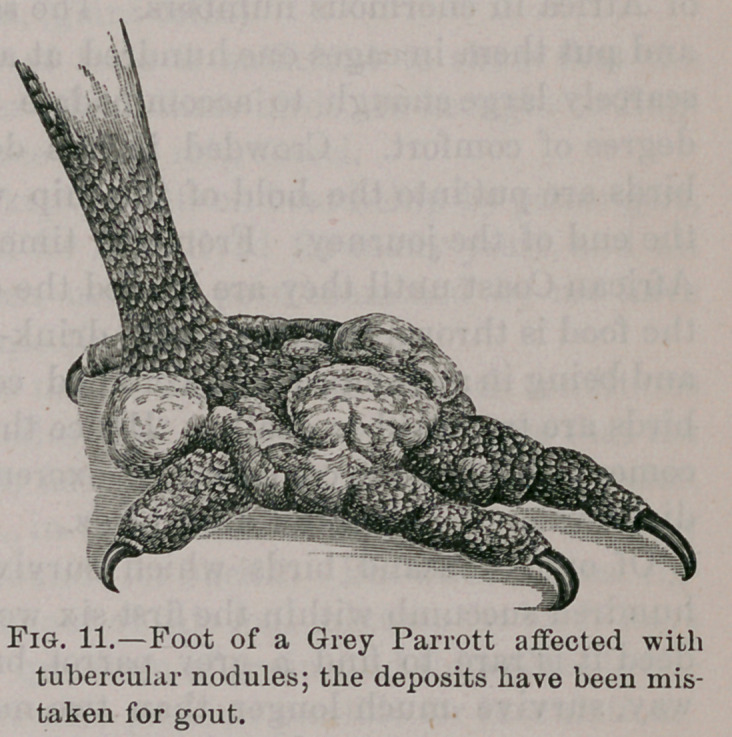# Avian Tuberculosis

**Published:** 1886-10

**Authors:** John Bland Sutton

**Affiliations:** Sir Erasmus Wilson Lecturer on Pathology, Royal College of Surgeons, England. Assistant Surgeon and Demonstrator of Anatomy, Middlesex Hospital, London


THE JOURNAL

OF

COMPARATIVE MEDICINE AND SURGERY.

VOL. VII. OCTOBER, 1886. No. 4.

### ORIGINAL COMMUNICATIONS.

### Art. XVI.—AVIAN TUBERCULOSIS. AN ILLUSTRATION OF AMŒBIC WARFARE.

BY JOHN BLAND SUTTON, F. R. C. S.

*Sir Erasmus Wilson Lecturer on Pathology, Royal College of Surgeons, England. Assistant Surgeon and Demonstrator of Anatomy, Middlesex Hospital, London.*

It is a fact well recognized that the inhabitants of certain regions of the earth suffer from diseases which are rare or even unknown in other parts, some affections are known to be endemic within even a limited area, whilst others are more or less peculiar to certain races of mankind independent of locality. Thus disease has an ethnological as well as a geographical distribution.

There is in addition a *zoological* distribution of disease, that is to say, every great group of animals suffers to a larger extent from some affections than others, these may not inaptly be termed the "scourges" of the group. Further if a disease is a scourge to two groups of animals, the lesions in both cases differ in their manifestations in important particulars. This may be partly due to structural peculiarities as well as to differences in the environment, whilst the specific irritant remains the same. The term "irritant" is employed to indicate any substance capable of initiating the inflammatory process. As we shall see later, the scourges are the result of the influence of *specific* irritants which have a predilection for certain groups of animals and, in some cases, for particular orders of a group.

There is also little doubt that the majority, if not all these scourges are due to the presence of living matter, either animal or vegetable, which thrives on the tissues of living forms. This is merely an extension of parasitism for as we know *Tænia echinococcus* is common to wolves and dogs, *T. medio cannellata* to sheep and oxen, *Cœnurus cerebralis* flourishes in the brain of sheep, and some species of *Acari* are only found on the ears of bats; these are merely familiar instances out of a long list that could be adduced.

To return to the affections or scourges with which we are most concerned in this article, it may be stated that syphilis, typhoid fever, and tuberculosis, (including in this latter term phthisis) are scourges of the human race. The first has never been found except in man. The second, typhoid fever* has been described in monkeys, and a few animals other than man. Tuberculosis though widely distributed, nevertheless devastates the human almost as extensively as any other species. Among the Equidæ glanders and anthrax are notorious affections, whilst asses are infested to an alarming extent by the worm *Strongylus armatus.* Walley speaks of Eczema epizootica, Perlsucht, Pleuro-pneumonia and Rinderpest as the four *bovine* scourges.

In this article it is proposed to give an account of a remarkable, widespread and fatal disease, affecting more particularly grain-eating birds, now becoming familiar as *Avian Tuberculosis.* The affection may be described as the scourge of this section of the feathered tribe.

### HISTORY.

Although several writers have described at different times isolated cases of tuberculosis in the fowl, it was impossible from the peculiarity of the lesions to decide whether the disease

*Path. Soc. Trans. vol. xxxvi, p. 527, and this Journal v. vii, p. 218.

was a genuine tuberculosis. Indeed it was not until the discovery by Koch of the tubercle-bacillus that pathologists had any definite criterion whereby the determination could be effected. So variously were the lesions in the case of birds interpreted, that as will be illustrated further on, some writers considered the lesions as scrofulous, others as gout, and in some instances they were reported as sarcomata.

My first researches into the nature of this disease commenced in the spring of 1879. A farmer having lost a large number of fowls in a very short time, requested me to investigate the cause of death, at the same time gave me permission to use the remaining birds in any manner likely to facilitate the inquiry. Taking full advantage of this opportunity I was enabled to make myself fully acquainted with the leading features of this interesting disease. In 1881 a second outbreak occurred which affected chiefly the young birds. In this as in the preceding epidemic only grain-eating birds were affected, ducks and geese escaped entirely. In the meantime fowls, from other poultry yards had been furnished me, and it soon became evident that the disease was widely spread in England, for specimens of the affection were received from Leeds and Middlesborough in Yorkshire, from Kent, very many places in Middlesex, and from Didcot and Hagbourne in Berkshire. The occurrence of tuberculosis in these places may be regarded as showing that it is probably met with in most parts of England.

In 1881 I began to attend systematically at the Zoological Gardens, London, and soon found the disease to be excessively common. For two years observations were made to determine the anatomical and zoological distribution of the disease, as determined by the analysis of the causes of death in one thousand birds of various species. In the meantime Dr. Heneage Gibbes had joined me in the work for the purpose of determining the relation of the bacilli to the lesions. In the following November (1883) we made a joint communication to the Pathological Society of London dealing briefly with the matter. Since that date many remarkable cases have come under observation which will enable a fairly complete monograph of this affection to be written.

During the early part of the investigation the view that the lesions were tubercular rested merely on analogy, but Koch's discovery of the tubercle-bacillus, in 1882, furnished an admirable means of judging as to the true nature of the nodular masses. It was for the purpose of establishing or disproving the tubercular nature of the disease that Dr. Heneage Gibbes in the summer of 1883 kindly took part in the investigation. The result was that myriads of bacilli were detected in the diseased tissues.

Before the results of our labors could be communicated to the Pathological Society, Ribbert of Bonn published a paper in the *Deutsche Med. Woch.* 1883, p. 413 and announced the parasitic characters of the disease and the identity of the bacillus with that described by Koch from diseased tissues in human tuberculosis. Since this date an admirable article has appeared in the *Journal de L'Anatomie et de la Physiologie*, Tom. xxi, 1885, written by V. Cornil et P. Mégnin, entitled "Mémoire sur la Tuberculose et la Diphthérie chez les Gallinacés," containing an account of the microscopical characters of the lesions, as well as confirming the presence of the bacilli. To all these investigators reference will be made in discussing the pathology of the disease, as well as to the inquiries of Nocard relative to the production of this disease, by feeding fowls with sputa derived from tuberculous patients.

Due credit must be given to Dr. O. Larcher, who, in 1871, published a short notice in the *Recueil Veterinaire*, headed "*Note pour servir a l'histoire de la tuberculisation du foie chez les oiseaux.*"

Dr. Edwards Crisp has recorded in the Transactions of the Pathological Society, London, several instances of tuberculosis in birds. A few isolated cases have been noted from time to time in various journals, but no systematic investigation, carried out on an extensive scale, has been attempted except my own endeavours. Again, it is necessary to emphasize the fact that we have no proof that these occasional cases reported as tubercle were genuine examples, for the disease is closely simulated by certain other affections, quite distinct in nature from tuberculosis, and it was not until the discovery of the bacillus, by Koch, that we had any criterion whereby differentiation could be effected with certainty. It is interesting that the identity of the bacillus, in avian and in human tuberculosis, should have been established at the same date quite independently by Dr. Gibbes and myself, in London, and by Dr. Ribbert at Bonn, although Ribbert's paper has certainly priority of publication.

As a matter of convenience a list of the chief writings relating to this disease is given here:—

1. LARCHER, Dr. O.—Note pour servir a l'histoire de la tuberculisation du foie chez les oiseaux.—*Recueil Veterinaire*, 1871.
2. CRISP, Dr. EDWARDS.—Some cases of tubercle in Birds scattered in the Transactions of the Pathological Society of London.
3. RIBBERT, Dr.—Ueber de Verbreitungsweise der Tuberkel bacillen bei den Hühnern.—*Deutsche Med. Wochenschrift*, July 11, 1883.
4. SUTTON, J. BLAND AND GIBBES, Dr. HENEAGE.—Tuberculosis in Birds. Read before the Path. Society of London, Nov. 1883. Published in *Path. Transactions*, vol. xxxv, 1884.
5. KOCH, Dr. R.—Die Aetiologie der Tuberkulose. *Mittheilungen aus dem Kaiserlichen Gesundheitsamte.* Herausgegeben von Dr. Struck. Zweiter Band, 1884, S. 1.

   This paper contains a brief account of tuberculosis in hens (Tuberkulose des Huhnes) at page 41 of the report.
6. CORNIL, V. and MEGNIN, P.—Memoire sur la Tuberculose et la Diphtérie chez les Gallinaces.—*Journal de l'Anatomie et de la Physiology.* Tom. xxi, 1885, p. 268.
7. LARCHER, Dr. O.—Etude sur la goutte des Oiseaux comparée a celle de l'Homme.

   A paper read before la Societé Central de Médecine Veterinaire, Jan., 1884.
8. KLEIN, Dr.—*Micro*-organisms in health and disease, a series of articles published in the *Practitioner*, in 1884. Since republished in a collected form.

   On page 101 of the first edition, the bacilli of a tubercular rhea are figured and described, taken from one of Dr. Heneage Gibbes' specimens.
9. NOCARD.—Sur une Tuberculose zoogléique des oiseaux de bassecour.—*Recueil de Médecine Veterinaire*, May 30, 1885.
10. NOCARD.—A paper on Tuberculosis in Birds, an abstract was published in the British Med. Journal, for July 10, 1886, taken from a paper read before the Central Veterinary Society of Paris.

Besides these paper, some important researches on infective diseases of birds possessing great interest in regard to the ætiology of these diseases are contained in the writings referred to below.

11. TRASBOT, LEOPOLD.—Expériences sur la transmissibilité de la Diphthérie des Volailles aux autres espéces animales. *Comptes Rendus de la Société de Biologie.* Series vii, Tom. 1, 1879.

12. MEGNIN.—Sur la Diphthérie des oiseaux et sa prétendue analogie avec celle de l'homme. *Comptes Rendus de la Société de Biologie.* Series vii, Tom. 1, 1879.

M. M. TRIPIER ET ARLOING—PIETRO PIANO.—Cornil and Megnin give an account with references to some independent researches of these writers on the subject of Diphtheria in birds and its relation to a gregarine. Consult the sixth reference in preceding list.

LŒFFLER.—Untersuchungen über die Bedeutung der Mikro-organismen für die Entstehung der Diphtherie beim Menschen, bei der Taube und beim Kalbe. Struck, *Mittheilungen.* Zweiter Band, 1884.

WOLFF, MAX.—Eine weitverbreitete thierische Mycose.—*Virchow's Archiv.* Band 92, S. 252.

### ZOOLOGICAL DISTRIBUTION OF THE DISEASE.

Zoological distribution of disease has received little attention at the hands of pathologists, yet many facts of great interest would be added to our stock of knowledge if this subject were systematically investigated. An inquiry into the distribution of tuberculosis among the various classes of the feathered tribe has been of signal service in enabling me to narrow the issue as to the probable origin of the disease. A somewhat detailed account may not be without interest to the reader.

In my early investigations note was made of the birds which suffered most from the disease, and it has been already mentioned, that so far as the farm-yard epidemics were concerned, that grain-eating birds were alone affected. Since I have been in regular attendance at the gardens of the Zoological Society, the average monthly mortality has been about one hundred, giving a yearly average for the past five years of one thousand, two hundred. Of this total nearly one-half (five hundred) were birds, and the list embraces most of those known to the ornithologist. From this source alone, a very good notion regarding the zoological distribution of the disease could be derived. Even after one year of work, it became evident that the disease was almost peculiar to graminiverous, and fruit-eating birds and vegetable feeders. The affection occasionally occurs in birds of prey, and its presence in them is capable of another explanation.

Birds which live on fish appear to be totally exempt from tuberculosis, and this is also true of water-fowl. Among struthionidæ, the rhea, (South American ostrich) is especially liable to the disease, indeed, among thirteen specimens of this bird which have died since 1881, twelve were thus affected, the remaining rhea was found dead one morning with a wound in the back of its head. It had only been an inmate of the gardens a few days, and was free from all traces of tubercular lesions.

The Emu *Dromæus novæ-hollandiæ* is subject to the affection *especially when born in the gardens.* The facts concerning these birds will be fully discussed in the section devoted to Pathology.

Of other species of birds, the common fowl, peacock, Guinea fowl, tragopan, grouse, pigeon and partridge are especially liable to tuberculosis. Storks and cranes are not exempt from it.

Opportunities have also occurred for studying the disease in parrots, and some facts of no small degree of importance have been brought to light by a careful examination of the circumstances in the outbreak of the disease in these birds.

With regard to the cases which have occurred in flesh-eating birds, a few remarks must now be offered. In the early account of avian tuberculosis, published in 1883, the only rapacious birds in which this affection had been detected with certainty were a falcon and an eagle, and it was added, "possibly they contracted the disease by feeding on smaller birds affected with tuberculosis." Since that date two other instances have occurred in flesh-eaters, an owl and the secretary bird. Diseases which simulate tuberculosis, especially so far as the liver is concerned, are often found in birds of prey, so that it is necessary to be careful in verifying the diagnosis by using the microscope. In the case of the secretary bird the characteristic nodules were observed in the mouth and on the tongue several weeks before it died.

The following observation made by J. F. Larcher shows that the disease may occur in wild birds, for he informs us in the note before referred to that a sparrow hawk which was taken in a snare at the moment when it pounced upon some gold finches, presented tubercular nodules in the liver, in the lungs and other parts of the body. Gold finches and other finches are frequently sufferers from this affection, and the hawk may have contracted the tubercles as a result of previous depredations among these pretty birds.

It is of utmost importance to adduce all the evidence that can be possibly pressed into the service, to strengthen the view that flesh-eaters get their tuberculosis by devouring infected grain-feeders, that the following case will be briefly quoted:

In February, 1883, I exhibited the liver of a Python, *Python sebæ* which presented curious nodules in the liver. The details of the case are reported in vol. XXXIV of the Path. Society's Transactions, and described as pyæmic abscesses, secondary to a large abscess in the wall of the abdomen, for it was difficult to account for them at that time in any other way that was satisfactory. As soon as bacilli were found in the nodules of the fowls, I at once submitted portions of the liver of the snake, which is preserved in the Museum of the Royal College of Surgeons to Dr. Heneage Gibbes to determine whether the bacilli which had been noticed in the lesions of the liver, were identical with those in the nodules of the bird's liver.

Examined under the improved knowledge derived from a study of the affection in birds, the case came out in a totally new light, for not only were the bacilli unmistakable tubercle-bacilli, but the microscopical details of the nodules in the snake's liver harmonized in every way with those characteristic of avian tuberculosis. Previous to this re-examination of the specimen by Dr. H. Gibbes and myself, Mr. F. Eve, the Curator of the Pathological Department of the Museum had rejected the pyæmic view of the lesions, and had deposited the specimen in the Museum as an example of tuberculosis. This independent testimony is very valuable.

This snake was fed on fowls, pigeons and ducks, as a rule, and there is, so far as I can see, only one way out of the difficulty. The source of mischief was undoubtedly derived from the alimentary canal; the birds, pigeons and fowls are excep-

*Fig. 6.*
*Section of the liver of a Pheasant affected with tuberculosis.*

*Fig. 4.*

*Duodenal loop of a Bird showing nodules of tubercle, N, N,. P. the pancreas. D.V. Duodenal Vein.*

*Fig. 7.*
*Spleen of a rhea affected with tuberculosis.*

*Fig. 1. Portion of Liver of a Python Python Sebœ, affected with tuberculosis.*

Lith. D. Appleton & Co. N.Y.

tionally liable to tuberculosis, and there can be but little doubt that this was the true source of infection.

When considering the enormous number of grain-eating birds which are affected, and the very few instances of the disease in fish and flesh-eaters, and bearing in mind the disease is an infective one, there remains little doubt on my mind that rapacious birds are affected through devouring tubercular prey.

In concluding this part it may be emphatically stated that, in the few isolated cases which have been recorded in periodical literature, the birds were graminiverous.

FIG. 1, Plate II.—Portion of the liver of a Python (*Python Sebæ*) affected with tubercle. The disease was probably contracted in consequence of the snake being fed on tubercular birds.

### ANATOMY OF THE ALIMENTARY CANAL.

Before entering into the details of the distribution of the lesions characteristic of avian tuberculosis, it is essential, for a clear understanding of the question, to consider the main features in the anatomy of the alimentary canal and especially in relation to the distribution and destination of the veins. It is unnecessary to discuss the various forms assumed by the digestive canal in birds, but to choose some type easily obtainable and of such a size that the individual parts may be demonstrated in a simple manner. The common fowl fulfills all the required conditions.

In the accompanying drawing (Fig. 2), *O* represents the œsophagus, presenting on the anterior aspect, near its middle, a globular swelling known as the crop, *C*. The œsophagus terminates in that singular muscular mill, the gizzard, *G*; from this organ the efferent canal is the duodenum, embracing the pancreas. The remarkable U-shaped arrangement of this part of the gut is peculiarly avian. Near the termination of the ascending or distal loop the bile and pancreatic ducts enter the wall of the bowel. To this succeeds the small intestines suspended, as usual, by a mesentery. Approaching the cloaca, two cæca (*Ca*) suddenly stand off at an acute angle. The point at which they join the gut marks the commencement of the large bowel; this, after a short course, terminates in the cloaca or common chamber for the genito-urinary products, *C*.

The most noteworthy features in the various sections of the alimentary tract, which have any bearing on the subject matter of this article, may be briefly mentioned. The proventriculus, or crop, possesses in its walls a series of glands, the secretion from which serves to moisten and soften the grain. The gizzard normally contains small stones, which are set in motion by the contraction of the walls and thus serve to triturate the grain. In many birds the mucous lining of the bowels has a soft, velvety appearance, due to the presence of innumerable villous processes. In no bird has the author yet succeeded in detecting Peyer's patches, although in a young Emu chick small rounded patches, not unlike the solitary follicles, were visible. The cæca vary in length within very wide limits. In the fowl they are six inches in length, yet in the pigeon they merely appear as a pair of minute diverticula from the wall of the bowel. The mesentery contains a plexus of lymphatic vessels of considerable size, but the mesenteric glands, so conspicuous in the majority of mammals, seem to be entirely absent in the bird. This is of some importance, for ignorance on this point has led one or two writers on avian diseases into a few misconceptions.

FIG. 2.—Alimentary canal of a fowl. *O*, æsophagus. *C*, the crop. *G*, the gizzard. *L*, liver. *Ca*, the cæca. *K*, the cloaca. *O*, the oviduct; and *P*, the pancreas.

We must now consider the disposition of the abdominal venous system. In birds a very free anastomosis exists between the portal and systemic veins; the venous circulation is further complicated by the existence, though slightly developed, of a renal-portal circulation. The arrangement of the veins may be gathered from a glance at the diagram, Fig. 3. The blood returns from the extreme hinder end of the body by the caudal vein, *C V*, which on reaching the first caudal vertebra divides into three main trunks, the lateral branch of each side is in the chick, reinforced by the hypogastric vein, and passes on to the kidney and distributes branches to that organ, which break up into a capillary venous network to join the radicles of the renal vein, thus constituting a rudimentary renal-portal system. The main stem of this vein joins the renal and is at this spot reinforced by the efferent vessel of the hind limb, the crural or sciatic vein. A common trunk thus arises which meets with the fellow of the opposite side to form the inferior vena cava. This receives various tributaries, but chief among them is the hepatic vein; it then opens into the right auricle of the heart.

Fig. 3.—A diagram to represent the relations of the portal and systemic circulations in birds. *C*, caudal vein, giving off *C V*, a connecting branch between the systemic and portal veins. *D*, duodenal vein, receiving branches from the gizzard. *S*, Splenic. *M*, mesenteric vein. *K*, the kidney. *H*, the liver. *V C*, vena cava inferior. *S*, sciatic vein.

Returning to the caudal vein we shall find that the median of the three trunks, into which it is divided, is of good size, and continues its course through the mesentery about half an inch from the border of the bowel. It forms not only an anastomosis between the portal and systemic veins, but may be regarded as the origin of the portal system itself. The vein follows closely the attached border of the gut and receives near the spot where it enters the liver, two tributary trunks of importance in connection with the distribution of the lesions. The two veins are the duodenal *D*, and the splenic *S*.

In mammals the portal trunk is usually the result of the confluence of the splenic and superior mesenteric veins. In birds, however, it may be regarded as being formed by the union of the duodenal and superior mesenteric veins, the splenic vein joining the trunk immediately above the union.

The splenic vein is of small size and commences in a few twigs on the walls of the proventriculus, these form a single vessel which runs along the hilum of the spleen.

The duodenal vein is formed by small branches arising on the wall of the loop, these converge so as to give rise to a median vein lying close to the pancreas, from which it also receives tributaries as well as numerous branches from the muscular walls of the gizzard.

The point of especial interest in this arrangement is, that if the portal circulation became hindered, congestion of the spleen would be a necessary consequence.

### MORBID ANATOMY.

The lesions peculiar to this disease differ in a remarkable manner from all known forms of tuberculosis, in that the alimentary canal and associated viscera are the parts almost exclusively affected.

In the first place the appearances found in typical cases will be described, then the chief variations and anomalies will be considered.

Birds which have succumbed to this disease are always extremely emaciated. On opening the abdomen a small quantity of fluid invariably escapes, for ascites is a prominent symptom in the later stages. The intestines are often matted together by adhesions and flakes of lymph are usually to be found. The most striking feature, however, is the existence of hard, irregular, craggy-looking masses, of a yellowish-white color, which project from beneath the serous coat of the small intestine. As a rule they are most abundant in the neighborhood of the duodenum and the segment of gut next succeeding. In severe cases the whole length of the alimentary canal, including the cæca, are studded with these nodules, which vary from a mass the size of a walnut to microscopic dimensions. The larger deposits are sessile, but it is by no means unusual to find small pedunculated masses of the size of a pin's head swinging from the exterior of the gut.

For a general notion of these refer to the duodenum shown in Fig. 4, Plate II. On cutting into the nodules they will be found to present a spurious capsule, but the interior is composed of pale-yellow material, exactly resembling that seen in a caseous abscess. On slitting up the gut it will be found that the condition of the peritoneal aspect is no guide to the state of the mucous membrane, for the interior will be found thickly studded with nodules in cases where only one or more of the masses can be detected externally. This is admirably illustrated in the case of the Emu whose intestine is represented in Fig. 5. The mucous surface is richly dotted with nodules but not one gave evidence of its presence externally. The nodules on the outer wall of the gut are of a pale color, and during life were covered with a rich plexus of capillaries, but those of the mucous surface are inky black in color, this is no doubt to be attributed to the action of sulphuretted hydrogen.

FIG. 4, Plate II.—The duodenal loop and pancreas of a fowl showing nodular tubercular masses.

The lymphatic vessels leading from the gut to the mesentery are often engorged with the same material which forms the centre of the nodules, and at times rupture and lead to extravasation of the caseous material between the layers of the mesentery; this has deceived some investigators—they describe such irregular masses as lymphatic glands, whereas these are absent in the bird's mesentery.

As might be expected the presence of these abnormal masses when of fair size cause intestinal obstruction, and a remarkable instance is represented in *Fig.* 5. It is the gut with the paired cæca of a rhea which died suddenly. On opening the abdomen, the peritoneal chamber lodging the intestines, was occupied by a large quantity of clotted blood. On removing the viscera the hæmorrhage was found to arise from some ruptured arterioles due to congestion as the result of an intussusception. The invagination commenced in the small bowel *S B*, in consequence of an annular tubercular mass deposited in its mucous coat *N′*, the lumen of the obstructed gut is indicated by the arrow. The shortening of the meso-cæcum *M*, as a result of the intussusception has allowed the cæcum *C′* to become also invaginated, assisted no doubt by the nodules $N''$. The length of the specimen is twenty-four inches. Smaller degrees of intussusception are very common. The cæca of the

FIG. 5.—Portion of the small and large intestine with the cæca of a rhea, *Rhea Americana.* The tubercular nodules have brought about intussusception of the small gut and led to shortening of the meso-cæcum so as to allow an invagination to occur in the right cæcum.

rhea presents an arrangement of its mucous membrane somewhat resembling the spiral valve found in Elasmobranch fishes.

FIG. 6, Plate II.—Section of liver of a Pheasant affected with tuberculosis. From a photograph by Dr. H. Gibbes.

FIG. 7, Plate II.—Section of the spleen of a Rhea affected with tuberculosis. From a photograph by Dr. H. Gibbes.

*The Liver.*—This gland presents remarkable changes; in severe cases it is enlarged and studded with rounded masses which may vary in size from a millet seed to that of a nut. When recent they are of yellowish-white color and often possess a very beautiful network of pink capillaries. On section they present the same caseous appearance as in the intestines. In the early stages it is impossible to accurately distinguish these deposits from those due to parasites such as gregarinidæ and the like, without searching for the bacilli. A typical example of a tubercular liver taken from a pheasant is shown in Fig. 6, Plate II, the nodules occur not only beneath the capsule of the liver, but throughout its parenchyma.

The *spleen* rarely if ever escapes, the deposits resemble in every respect those met with in the liver and intestines. A severe example of the disease affecting the spleen is shown in Fig. 7, Plate II, taken from a rhea, *Rhea Americana.* In some cases the spleen is so crammed with the morbid material that the capsule ruptures.

The lungs are not often affected and when the nodules are present in these organs they rarely exceed in size a split pea.

The larynx I have seen affected twice only, in a secretary bird and a hornbill.

*Lymphatic System.*—It has already been mentioned that the lymphatic vessels of the mesentery become engorged with caseous material. In severe cases, the lymphatics may become generally affected and the morbid material dispersed throughout the body—the eyelids, wings, thighs and subcutaneous tissues. Two lymphatic glands, situated in many birds at the root of the neck, may occasionally become infected and may attain a very large size.

A careful comparison and analysis of many cases of tuberculosis in birds, made at all stages of the disease, enables me to offer a satisfactory explanation of various apparant anomalies in the distribution of the lesions.

In the first place, it will be assumed that the morbid material gains entrance, in the majority of cases, by way of the intestines. In every case examined by me, in which the disease originated in this way, the duodenum has always been affected and has been found diseased when no other deposits were visible. The duodenal vein is of large size (Fig. 3, page 339), opens into the portal vein and goes at once to the liver, which is the second viscus in order of infection. The disease extends along the gut; meanwhile, the deposits in the liver, increasing in number and dimensions, necessarily hinder the portal circulation and retard the flow of blood from the spleen. This damming back of the splenic blood allows the infective bacilli to find an easy road to the spleen, and this organ is the third in order of infection.

The majority of cases never pass beyond this stage; occasionally, however, the bird survives longer, and the gut becomes further involved, until the region of the bowel drained by the vein marked *C V*, in Fig. 3, is infected. As soon as this occurs, the morbid material from the large bowel takes a reverse course and passes to the kidney, infects them, then flows to the inferior vena cava, enters the heart, and becomes disseminated everywhere. So long as the upper segment of the gut alone is affected the capillaries of the liver act as efficient strainers, and arrest the wandering invaders; but as soon as the lower segment is reached, and the infected blood passes by way of the systemic veins, universal distribution is the result.

But this, though by far the most common, is certainly not the only means by which the disease is acquired by birds. In discussing the question of the probable origin of the affection, some remarkable examples of inoculation, by way of the feet, will be considered.

*Microscopical Details.*—The histology of the tissues affected in this disease demand consideration under three headings. (1) The structure of the nodules. (2) The characters of the bacilli and their relation to the lesions. (3) The blood vessels and the share they take in distributing the bacilli.

(1). *The Structure of the Nodules.*—It is preferable to choose portions of diseased tissue from the liver for purposes of examination, and to classify the nodules into two groups, recent and old, for they differ materially in structure and relation to bacilli according to their age.

In the youngest nodules the centre is made up of small round, and giant cells, whilst the periphery is made of fibroid material, such as is usual in the immediate neighborhood of inflammatory foci. The bacilli are clustered together in the centre of the mass, and many of them occupy the interior of the cells; sometimes as many as three or four bacilli may be counted in a single cell. In the giant cells as many as sixty have been counted. Cornil and Megnin describe a specimen of the bacilli, taken from the liver of a pheasant, as being arranged in the form of a crown. In some cases the bacilli are seen crowded in little heaps, and, in others, arranged in tubular masses as though occupying the lumen of a vessel.

In nodules slightly older the centre is occupied by a more or less caseous mass, quite free from bacilli, these little bodies occupying a zone outside the degenerating area.

In successful sections of the largest nodules four zones may be distinguished. The centre is occupied by a caseous material; this is bounded by a ring of cells, resembling those found in human tuberculosis, and termed "epithelioid;" to this succeeds a layer which may be named, with Ribbert, "the bacilliferous zone," and, lastly, we have a zone of inflammatory tissue. In the bacilliferous zones giant cells may be found of very large size, and frequently four or five may be counted in a single section.

There is substantial agreement on all points connected with the histology of the nodules in the account given by Ribbert, Cornil and Megnin, Heneage Gibbes and myself. The relation of the bacilli to the cells are shown in Fig. 8. Cornil and Megnin have given some drawings from which a very good notion may be formed of the enormous number of bacilli which crowd the sections, especially those made from the liver. The bacilli may be stained by any of the ordinary re-agents used for detecting them in human sputum.

(2). *The Bacilli.*—All who have inquired into the subject of avian tuberculosis in reference to the bacillus have come to the conclusion that it is identical with Koch's tubercle-baci[illegible]-

lus, not only in its morphological characters, but also in its re-action to staining reagents, Dr. Gibbes * reports as follows:— "They have the same re-action to staining agents as the bacilli of tuberculosis, with a high magnifying power (x 4000) they are indistinguishable from them, and they also contain rounded bodies resembling spores." Cornil and Megnin draw attention to the fact that some of the cells present granules in their interior, which take the same stain as the bacilli. They also mention that the bacilli contain granules in their interior, visible under high magnifying power, and add: "The large cells bristling with tufts of bacilli usually possess a single nucleus, they resemble leprosy cells, but contain more bacilli than is usual in the large cells of leprosy nodules." Dr. Klein † who had an opportunity of examining some of the sections from the liver of the rhea, regarded the disease as leprosy;

FIG. 8.—*A*, a section of an early tubercular nodule from the liver of a pheasant with caseating centre. *B*, section of a nodule in a later stage, to show the caseous center and the marginal bacilliferous zone.

this is obviously a mistake, the two affections having little in common, but a resemblance of bacilli.

(3). *The blood vessels considered in relation to the distribution of the bacilli.*—From an anatomical standpoint alone it is evident, as I have endeavored to show, that the bacilli are distributed by means of the blood vessels. Whilst detailing the microscopic appearances of the nodules, attention was drawn to the fact that the bacilli are in places crowded in little heaps, and in places arranged in tubular masses as though occupying the lumen of a vessel.

* Path. Trans. vol. xxxv.

† Micro-organisms and disease. A series of articles in the *Practioner*, 1884.

Cornil and Megnin note that in some of the section the bacilli lie crowded in fissures, some of which represent vessels cut longitudinally. In one of the drawings taken from the liver of a pheasant a vessel is shown in tranverse section, the lumen of which contains a cluster of these little rods.

If any one takes the trouble to examine the liver in the earliest stage of tuberculosis, or endeavor to select recent nodules in an infected liver, he will be able to satisfy himself that in the majority of cases the nodule is in close association with a vessel, and in very many cases the lumen of the vessel forms a central spot from which the bacilli radiate as from a focus.

The best research on this head is that of Ribbert, who studied this part of the matter in the pulmonary vessels, for he found the parallel bundles of connective tissue forming the walls of the veins thickly invaded by the bacilli which were grouped together in irregular masses. In places they encroached upon the lining membrane of the vessel and caused bulgings resembling minute tubercles and similar relations could be made out on the arteries.

Fig. 9.—Section of a vein from the lung, the walls of which are crowded with tubercle-bacilli.

(4). *The relations of the blood to the bacilli.*—Within the last few years the attention of biologists has been attracted to the phenomenon known as intra-cellular digestion. Chief among investigators in this particular direction is the eminent Russian naturalist, Metschnikoff, who has published accounts of most interesting combats he has witnessed in invertebrate forms between bacilli and leucocytes. These researches have gone a very long way towards modifying our views of the nature of the inflammatory process. In my lectures delivered at the Royal College of Surgeons, London, in the capacity of Erasmus Wilson Lecturer, and since published in a collected form,* a review of the chief facts connected with this important phenomenon is given in its bearing on the pathology of inflammation. It is not proposed to spend time in discussing

* An Introduction to General Pathology, 1886.

the evidence in this paper, but I shall be content with observing that there are grounds enough for believing that when bacilli, bacteria, micrococci and other micro-organisms enter animal bodies, the leucocytes, (if the organism is sufficiently complex to possess such bodies,) engage them in combat and attempt their destruction by ingesting and digesting them, and so rid the body of substances calculated to do it harm.

My studies on avian tuberculosis have convinced me more thoroughly of the defending power exercised by leucocytes than any observations I have yet been able to make on an animal so highly organized and specialized as a bird.

There can be no doubt that the bacilli in this affection from whatever source arising, are introduced with the food into the alimentary canal, and thence find their way into the walls of the bowels.

That the bacilli gain entrance to, and are disseminated by the blood vessels is beyond all question, as they have been found by those who have investigated the disease occupying the interior as well as the walls of arteries and veins.

On reaching the vessels the bacilli are attacked by the leucocytes, surrounded and taken into the protoplasm entire, where they may become digested and destroyed. One leucocyte may ingest two or more of the enemy.

The leucocytes when laden with spoil need not necessarily remain in the vessel but pass through the vessel-wall into the surrounding tissue. Further from what we now know of "amœbic warfare" in invertebrates, frogs, rabbits, etc., if a detachment of bacilli occupy a capillary or leave the vascular channels, a troop of leucocytes are quickly on the spot and become reinforced by comrades at a rate wholly out of proportion to the ratio normally existing between the red and white cells of the blood. Metschnikoff has shown and I have confirmed his results, that in some cases a leucocyte struggling with a bacillus or a coil of bacilli, may receive help by two or more leucocytes fusing with it to form a larger mass—a giant cell. In the lower animals giant cells occur with much greater frequency than in man, in lesions, which are the result of specific irritation, that is to say, due to the introduction into the system of a virus, such as glanders, actinomycosis, syphilis, and the like. The lesions characteristic of this class of affections belong to an increasing list known as the infective granulomata, whose chief characters consist of the following: They are made up chiefly of round cells with very little intercellular material, in most instances giant cells are present; they are poorly supplied with vessels, hence the central portions, those farthest removed from the blood stream die. Micro-organisms of some kind or another are usually to be detected, and the list of lesions of this class in which they have not yet been found is rapidly becoming reduced to insignificant proportions.

In actual war the victorious, as well as the defeated army, lose numbers of soldiers. In amœbic warfare the bacilli may gain the victory either by force of numbers or by their poisonous effects upon the leucocytes. In tuberculosis the characteristic nodules are to be regarded as battle fields. In the small, recent nodules the struggle was raging at the time the bird died. In the later nodules the central caseous mass is composed of the dead and dying cells, some of which are undergoing disintegration whilst the combat is still going on along the borders. The largest masses mark the spots where the conflict has been long continued. It is in these older masses that the giant cells most frequently occur and as in other cases they arise from the fusion of leucocytes. The giant cells are powerful defenders, for in some nodules taken from the liver of a rhea as many as fifty bacilli could be counted in a single section of one of these singular bodies. In such specimens as these the mass when stained becomes visible to the naked eye, for the bacilli take up the stain so as to give rise to the appearance of a colored dot.

The observed facts in this disease show that it is a struggle between irritant-particles and leucocytes,—in essence, amœbic warfare.

### ÆTIOLOGY.

The ætiology of avian tuberculosis is in very many respects one of great importance, for if we arrive at a satisfactory conclusion on this head we may assume with some degree of probability the origin of human tuberculosis also. This section, on these grounds alone deserves the fullest consideration. The zoological distribution of the disease shows it to be almost exclusively confined to grain and vegetable feeding birds, whilst the localization of the lesions convincingly demonstrate that the bacilli are introduced into the system by way of the alimentary canal and thence distributed by the portal and systemic veins.

In the Zoological Society's Gardens, London, the opportunities most favorable for investigation of this question occurred in the pheasants, rheas and emus. The pheasants have for years occupied a particular portion of the gardens, arranged so as to constitute a series of wire enclosures known as the pheasant's aviary. The rheas and emus occupy a series of sheds with adjoining paddocks, and are close neighbors. The ostriches and cassowaries are kept in a part of the menagerie quite distinct from the rheas and emus.

It seemed desirable in the first place to submit the food and water taken by these birds (maize, and monkey-nuts for the pheasants, vegetables and boiled maize for the rheas and emus) to microscopical examination for the purpose of ascertaining the existence of bacilli, but all to no purpose.

That the birds contract the disease in the gardens is beyond doubt. All the rheas which have come under my observation have been imported, some had lived many years in the gardens.

In March, 1884, two young emu chicks were hatched; for a time they did well. In March, 1885, one died and on examining the alimentary canal tiny nodules such as are represented in Fig. 10, were found dotting the mucous membrane.

The nodules contain chiefly round cells of various sizes, here and there giant cells and crowds of bacilli. If they be attentively examined the larger ones present a caseous interior and in many instances the caseous matter has burst the capsule and presented as a black mass looking like a miniature cutaneous horn growing from a sebaceous cyst. One of these masses magnified three times is shown in Fig. 10. The liver and spleen presented the characteristic deposits.

Three months after, the companion chick died and an examination of its viscera showed the nodules in a more advanced stage than in the first chick.

For the sake of comparison a piece of gut from this bird is represented by the side of the earlier specimen.

After the caseous matter bursts the capsules it doubtless increases in size from deposit of fæcal matter from the bowel.

These specimens help to show that the disease originates in the mucous coat of the bowel. Recently an emu died which had only lived in the garden seven months, yet its alimentary canal presented nodules such as are seen in *B*, Fig. 10. The specimen is valuable as showing the chronic progress of the disease.

FIG. 10.—*A*, tubercular deposits in the alimentary canal of an emu—early stage. *B*, a later stage. (*C*) one of the nodules from *A*, magnified three times to show the curious horn-like appearance it presents.

The nodules serve as "breeding-grounds" wherein the bacilli may be propagated and the experiments of Cornil and Megnin show that the masses are quite capable of instituting the disease. They injected caseous matter from this source into the peritoneal cavity of a fowl and a guinea-pig. The fowl died some months after with the disease in its liver and intestines, whilst the guinea-pig presented a large caseous abscess at the seat of puncture containing an astonishing number of bacilli.

Although the nodules in the alimentary canal contain thousands of bacilli and in spite of the almost positive evidence that the disease is introduced into the system with the food, yet bacilli have as yet not been detected in the fæces. This, however, does not militate against the bacilli being introduced with the food, for it is not to be supposed that bacilli are always being swallowed; probably one inoculation is sufficient and the micro-organisms once in the body find it a suitable place for indefinite multiplication. There is one other important matter in connection with the fæces, demanding full consideration. The farm which originally supplied me with tubercular chickens and fowls, is now entirely rid of the disease. Following my advice the old stock of birds were entirely replaced by new ones. The hen-houses were cleaned, the old soil replaced by new, and a limited number of birds allowed to roost in each house, the walls and roosts of which are periodically limewhited, and the floors cleaned regularly.

Before these precautions were taken birds died almost daily, but now the disease has entirely disappeared. If we inquire closely into the manner adopted in feeding birds of this class, we shall find that the seed is cast upon the ground to be picked up grain by grain. The birds are constantly dropping their excrement over the same ground, so that the ingesta and egesta are mixed together, in this way the food of to-day is contaminated with fæces which may be many months old. If the birds are confined in paddocks or wire enclosures, the upper layers of the soil must be thoroughly saturated with a mixture of stale food and fæces. It is to this that we must regard as the cultivation material for the bacilli.

Although I made attempts to search for bacilli in these masses of fæces, and have kept birds excreta in variable temperature for long periods, I have as yet failed to detect the bacilli, however, the negative results by no means disproves fæces, and food remains being the growing nidus for these bodies.

The following case seems to me to support the notion of the disease originating in this way very strongly.

Parrots, especially grey ones, have occasionally come to hand with caseous nodules developed upon their feet, resembling in structure those found in the alimentary canal. They also contain colonies of bacilli. In other cases these nodules occur on the wings and trunk, but the viscera remain healthy. The appearance of the feet when affected with tuberculosis may be inferred from Fig. 11. An eminent London physician to whom some of the nodules were shown declared it to be gout.

Dr. O. Larcher published a memoir entitled '*Etude sur la goutte des oiseaux comparée a celle de l'Homme,** but a perusal of the paper and all the cases he refers to and describes under the heading of generalized gout, visceral gout, and arthritic gout, are examples of the tuberculosis we have been considering. Some of the instances mentioned by Larcher occurred in parrots and parrakeets. My own investigations in this direction have served to convince me that these so-called gouty deposits are tubercular nodules which have had a local origin, and that they have been caused by the birds hopping about in the mixture at the bottom of their cages, which is composed of remnants of food, water and a very large admixture of fæces. These cases strongly support the argument that the true source of the bacilli is in this material.

FIG. 11.—Foot of a Grey Parrott affected with tubercular nodules; the deposits have been mistaken for gout.

Grey parrots are very liable to fall victims to an affection the result of micro-coccus invasion, the organisms causing the disease in this instance originating in the fæces and remains of food.

* Read before la Societé Centrale de Médecine Veterinaire, Jan. 1884.

That the majority of grey parrots brought to England die within six weeks of their importation is a fact notorious in itself, and the reasons have been plainly set forth in a paper by Max Wolff,* headed, *Eine weitverbreitete thierische Mycose.* All the facts stated in Wolff's paper I can confirm from my own experience, as from time to time many of these parrots have come into my hands direct from the dealers in Liverpool, also from various private sources.

Grey parrots are brought to this country from the West Coast of Africa in enormous numbers. The sailors buy the birds and put them in cages one hundred at a time, the cages being scarcely large enough to accommodate twenty-five with any degree of comfort. Crowded in this detestable fashion the birds are put into the hold of the ship where they remain to the end of the journey. From the time the birds leave the African Coast until they are landed the cage is never cleaned; the food is thrown to them, their drink-water rarely changed, and being in a cramped and confined condition some of the birds are trampled to death. Hence the floor of the cage becomes a filthy mass, composed of excrement, remains of food, dirty water, feathers and dead birds.

Of one thousand birds which survive this journey, eight hundred succumb within the first six weeks of landing. Indeed it is rare to find a grey parrot, brought home in this way, survive much longer than two months. The chief appearances found in birds dying in this way, are the following:

The liver contains some two or three score of greyish-white nodules, varying in size from a pin's point to that of a pea or larger. These masses though most numerous, immediately subjacent to the serous membrane are nevertheless scattered throughout the hepatic parenchyma. The spleen and kidneys are similarly affected, the former far more frequently than the latter organ. The lungs present similar lesions in about twenty per cent of the cases examined.

If sections of the nodules be examined after staining with methyl violet, gentian violet, or aniline brown and magnified, heaps of micro-organisms collected in heaps and lying for the most part in the capillaries will be detected. In some cases

*Virchow's Archiv. Bd. 92 p, 252.

these micro-organisms so completely block the vessels as to produce thrombosis and ectasia.

The micrococci, when examined under a Zeiss' oil immersion one-twelfth, ocular 4, are seen as rounded bodies; sometimes they are ovoid in appearance and one-twelfth of a millemetre in diameter; some are twice that size. Suppuration is absent even in the largest nodules. In some of the nodules I have found numbers of giant cells, as many as five or six could be counted in a single section. Micrococci could be detected in some of the plasmodia, (giant-cells.)

The brief narration of facts is sufficient to show that the filthy conditions under which these birds are brought to England explains the source of the micrococci.

Many grey parrots kept as pets on board ship by passengers, properly fed and cared for have lived for many years, and all birds which have been used as pets, examined by me have had no traces of the infective little organisms.

If micrococci of this nature multiply under such conditions and invade animal bodies need we hesitate to believe that the gradual, but constant, accumulation of fæces, food remains, moisture, feathers, &c., in farm-yards or in a crowded hennery should be breeding grounds for bacilli. But what is most significant, is the fact that out of this foul mixture the birds have to pick up their daily quantum of grain, and thus place themselves in the most favorable position possible for inoculation.

The latest contribution to the ætiology of this affection is by Nocard in a memoir read before the Central Veterinary Society, of Paris.* This writer has investigated four epidemics in poultry yards and has come to the conclusion that the birds contracted the disease by coming in contact with persons in the last stage of phthisis. In another epidemic he believed the disease to have been caused by the bird feeding in yards contaminated with the excrement of tuberculous cows. These views are highly improbable.

Nocard, however, is to be congratulated on the result of his experiments for he has succeeded in inoculating tuberculosis from birds to mammals, and conversely has shown that avian tuberculosis is transmissible to mammals. He has obtained

* See an abstract in the British Medical Journal, July 10, 1886.

successful results with several animals, among them rabbits, guinea-pigs and kids. Development of the disease was very slow, three to five months being usually required for the purpose. This, I believe, to be a fair estimate.

The most interesting part of Nocard's work is, that he has succeeded in cultivating the bacillus in gelatinized serum and has obtained bacilli up to an eight cultivation. His method of procedure differs slightly from that employed by Koch, in that he obtains the serum direct from the jugular vein; this renders sterilization unnecessary.

CONCLUSIONS.

A critical study of the ætiology of avian tuberculosis suggests some very important conclusions which have a great bearing on the probable source of the bacilli which infect human beings. Indeed, it is not improbable that the tubercle-bacillus may have its origin in this way. The statements of Nocard, given on the preceding page, that, in the epidemics investigated by him, the assumed cause was inoculation from the sputa of phthisical patients, will not bear the test of criticism.

All who have inquired into the lesions of tuberculosis have been struck by the myriads of bacilli present in the lesions; this is sufficient to show that the tissues of birds are especially favorable for the growth of this micro-organism. Those who have cultivated the tubercle-bacillus are unanimous in the opinion that it thrives best at a temperature varying between 37° and 39° C. The average temperature of a fowl, taken in the cloaca, is about 40° C, and this increase of temperature over the average human body-heat may explain the luxuriant growth of bacilli.

When microscopists shall have exhausted their endeavors in detecting specific differences among the infinite variety of micro-organisms, and then direct their endeavors towards the phylogeny of the various forms, they will possibly find that this remarkable bacillus, which may fitly be termed, with typhoid fever, a scourge and a bane of civilization, has been evolved from some harmless form, and that its peculiar character of thriving best at a temperature equal at least to that of the human body, was first acquired by being transplanted into a medium, the chief constituents being *animal excreta.*

I feel confident that, if due attention be directed to the disease known as "avian tuberculosis" in various parts of the world, especially with regard to its ætiology, we shall come to the conclusion that tuberculosis will stand a good chance of becoming included in that important list of diseases communicated to man from the lower animals. The list of fatal cases of tuberculosis occurring in man, appalling though it be, is small compared to the relative mortality, from this disease, among the grain-eating members of the feathered tribe.

## Figures and Tables

**Fig. 1. f1:**
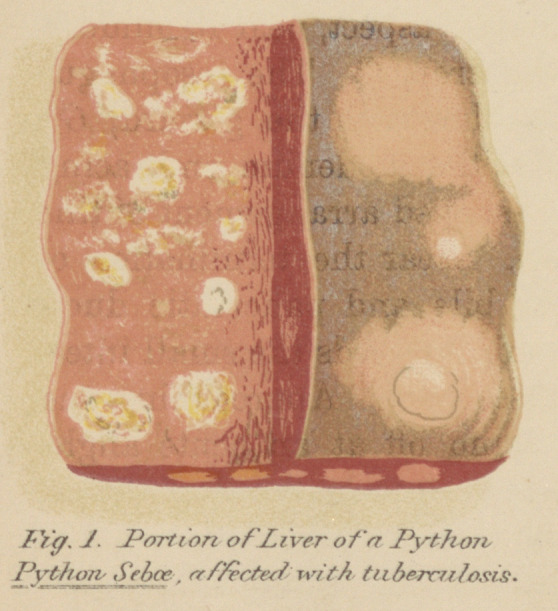


**Fig. 2. f2:**
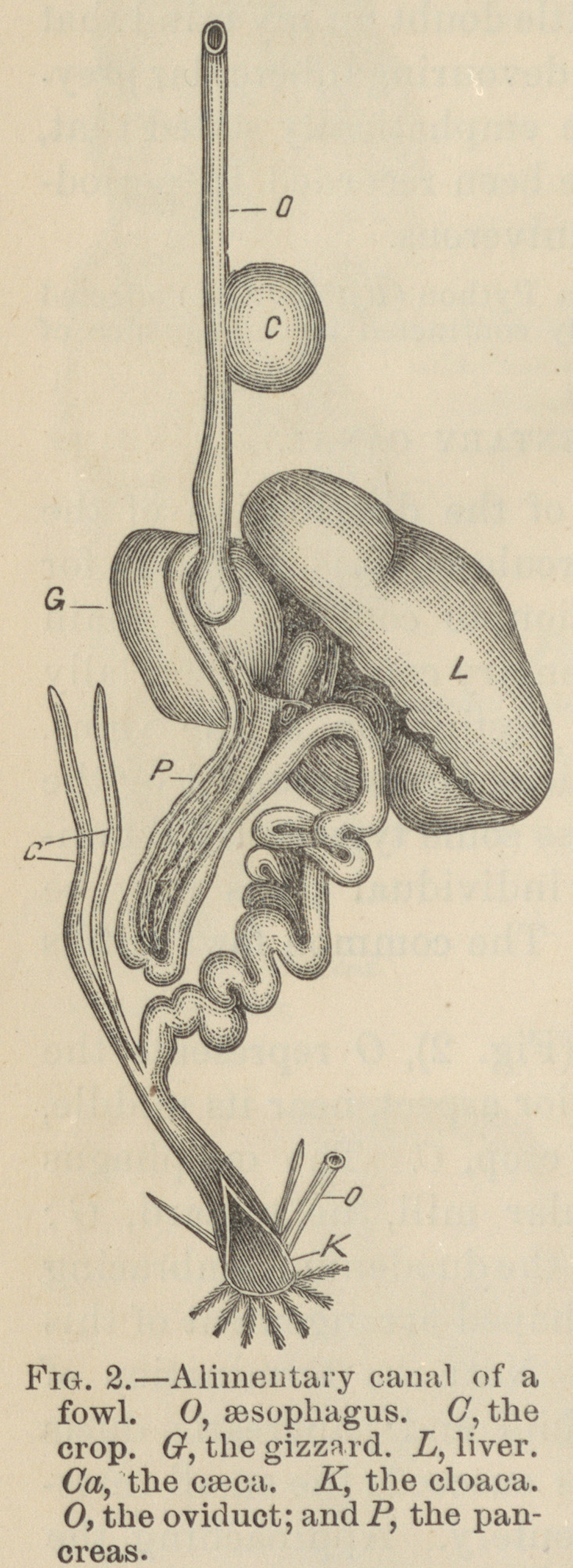


**Fig. 3. f3:**
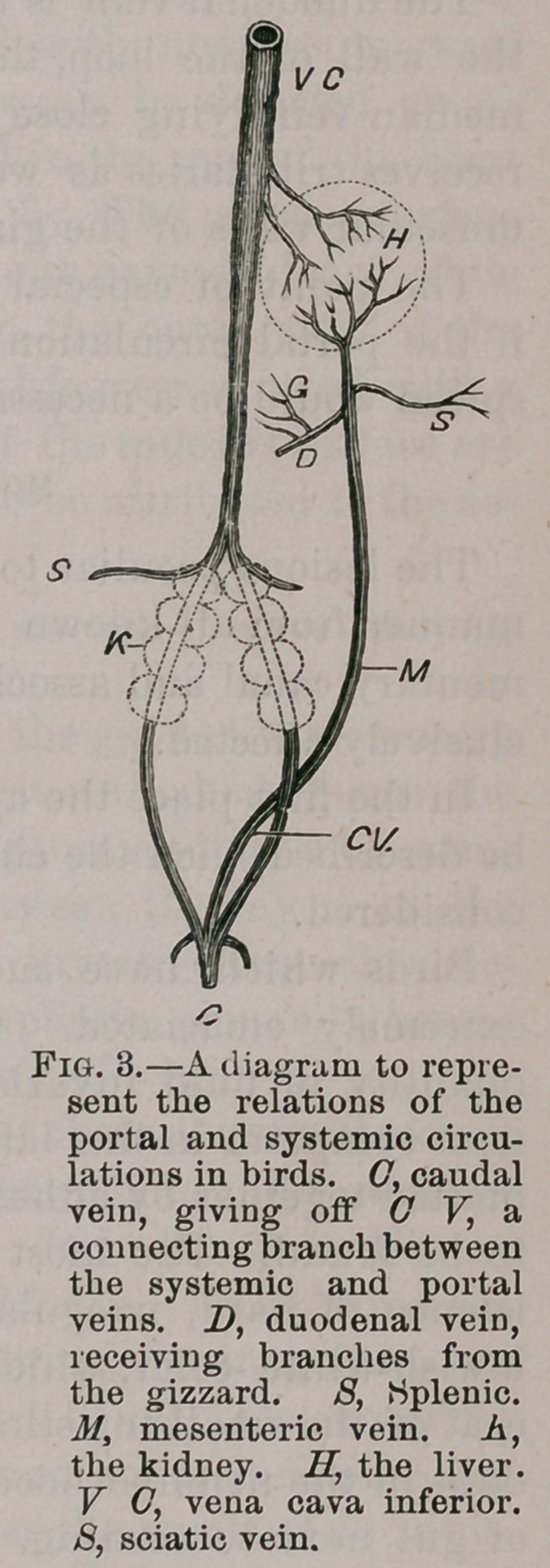


**Fig. 4. f4:**
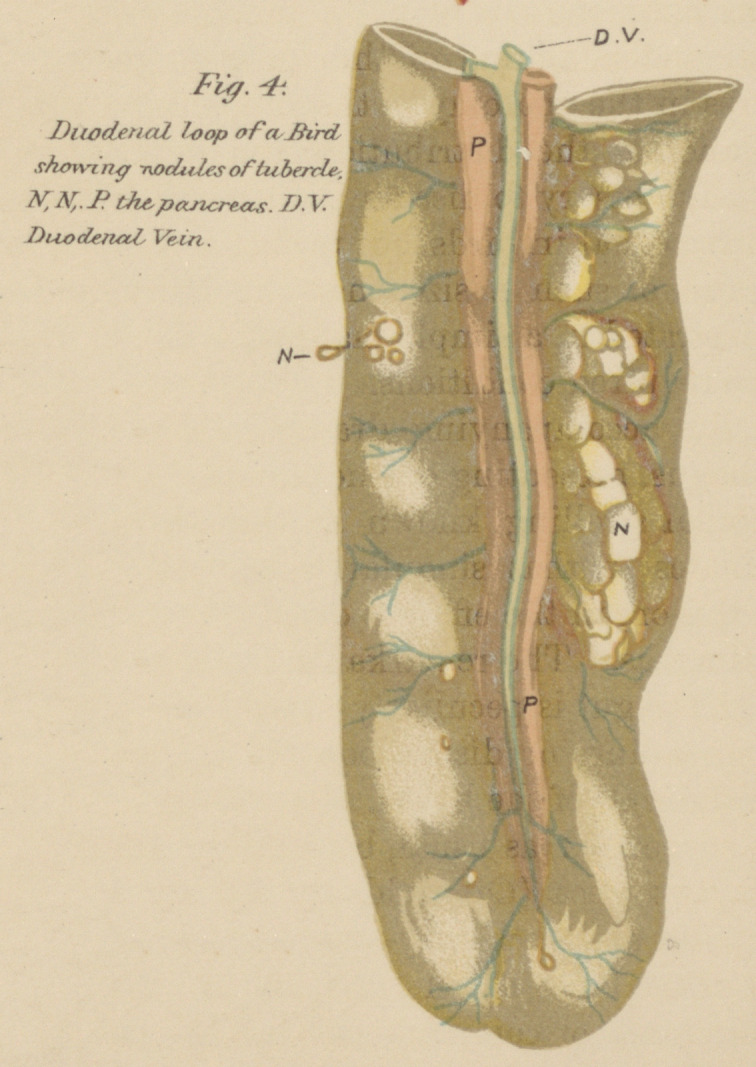


**Fig. 5. f5:**
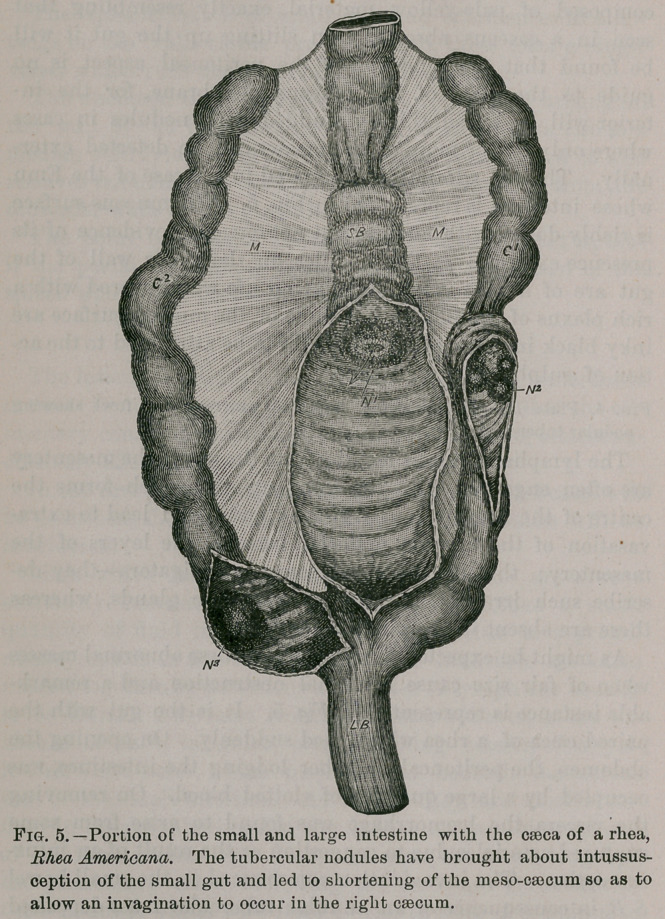


**Fig.6. f6:**
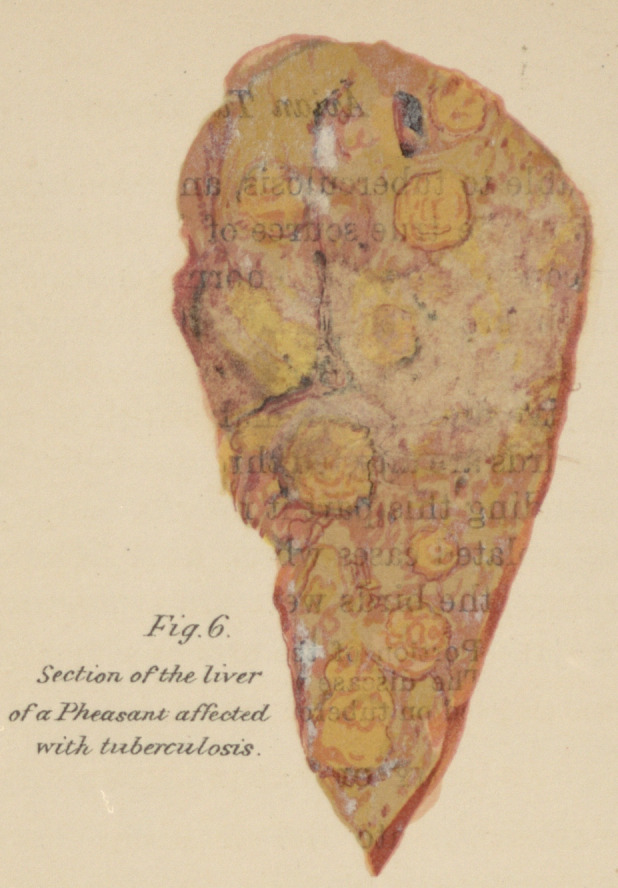


**Fig.7. f7:**
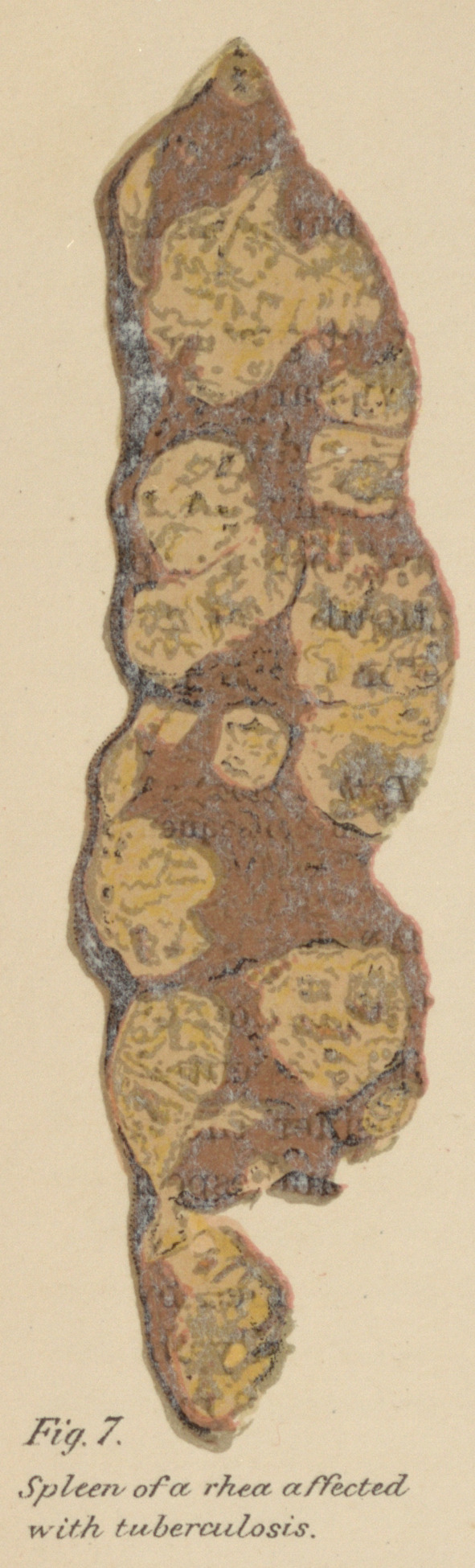


**Fig. 8. f8:**
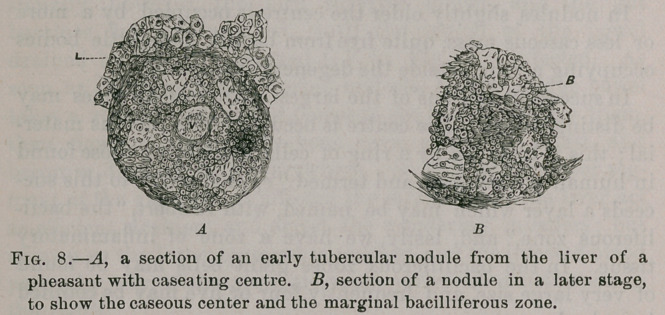


**Fig. 9. f9:**
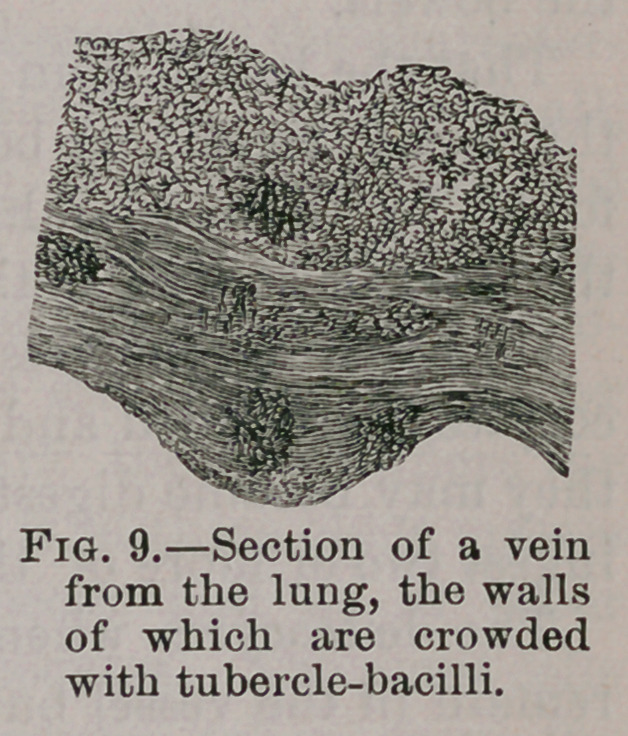


**Fig. 10. f10:**
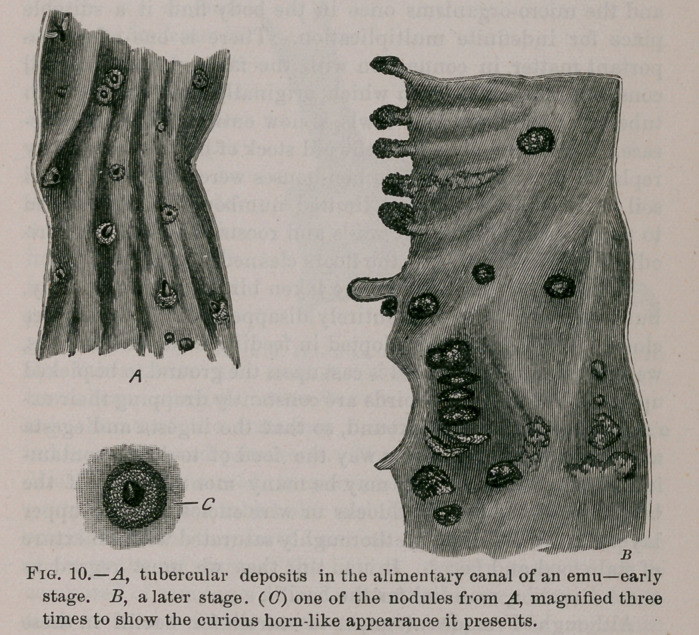


**Fig. 11. f11:**